# Supplementary material for: The transcription factor Pou3f1 promotes neural fate commitment via activation of neural lineage genes and inhibition of external signaling pathways
Source: eLife. 2014 Jun 14;3:e02224. doi: 10.7554/eLife.02224 (PMC4095939; doi:10.7554/eLife.02224)
Supplement: Supplementary file 1. — Primer list for PCR analysis. (A) The PCR primers used to clone Pou3f1 into the lentiviral expression vector pFUGW-IRES-EGFP. (B) Oligo sequences used for Pou3f1 RNAi. (C) Primers used for Real-time Q-PCR analysis. (D) Primers used for ChIP-qPCR. DOI: http://dx.doi.org/10.7554/eLife.02224.018 [file elife02224s001.docx]

**Supplementary File 1**

| 1. **Primers for mPou3f1-IRES2-EGFP** | | |
| --- | --- | --- |
| F: 5’-cggGAATTCATGGCCACCACCGCGCAGTAT-3’ | | |
| R: 5’-cggGGATCCTCACTGCACAGAGCCGGGCA-3’ | | |
| 1. **Oligo sequences for Pou3f1 RNAi** | | |
| control-RNAi FOR | gatccccCTTGTGAAGCTTCTAGGAGttcaagagaCTCCTAGAAGCTTCACAAGtttttggaaa | |
| control-RNAi REV | agcttttccaaaaaCTTGTGAAGCTTCTAGGAGtctcttgaaCTCCTAGAAGCTTCACAAGggg | |
| mPou3f1-RNAi-1 FOR | gatccccGCAGCGGAAGATCCAGAATttcaagagaATTCTGGATCTTCCGCTGCtttttggaaa | |
| mPou3f1-RNAi-1 REV | agcttttccaaaaaGCAGCGGAAGATCCAGAATtctcttgaaATTCTGGATCTTCCGCTGCggg | |
| mPou3f1-RNAi-3 FOR | gatccccGCAGGAAATAGCCAAAGCTttcaagagaAGCTTTGGCTATTTCCTGCtttttggaaa | |
| mPou3f1-RNAi-3 REV | agcttttccaaaaaGCAGGAAATAGCCAAAGCTtctcttgaaAGCTTTGGCTATTTCCTGCggg | |
| 1. **Primers for Real-time Q-PCR** | | |
| Gene | Forward primer （5′ to 3′） | Reverse primer （5′ to 3′） |
| mPou3f1 | TCGAGGTGGGTGTCAAAGG | GGCGCATAAACGTCGTCCA |
| mRex1 | CAGTTCGTCCATCTAAAAAGGGA | TCTTAGCTGCTTCCTTGAACAATGCC |
| mNanog | TTGCTTACAAGGGTCTGCTACT | ACTGGTAGAAGAATCAGGGCT |
| mOct4 | CAGAAGAGGATCACCTTGGG | GTGAGTGATCTGCTGTAGGG |
| mFgf5 | GCTGTGTCTCAGGGGATTGT | CACTCTCGGCCTGTCTTTTC |
| mSox1 | TGCAGGAGGCACAGCTGGCCTAC | TGCCGCCACCGCCGAGTTCTGG |
| mSox2 | GCGGAGTGGAAACTTTTGTCC | CGGGAAGCGTGTACTTATCCTT |
| mPax6 | GCAGATGCAAAAGTCCAGGTG | CAGGTTGCGAAGAACTCTGTTT |
| mZfp521 | GAGCGAAGAGGAGTTTTTGG | AGTTCCAAGGTGGAGGTCAC |
| mNestin | GCTGGAACAGAGATTGGAAGG | CCAGGATCTGAGCGATCTGAC |
| mZic1 | AGAGCAGAGCAACCACATCT | CCCCTGTGTGTGTCCTTTTG |
| mZic2 | AGTGTGAGTTCGAGGGCTGT | GGGATGCGTGTAGGACTTGT |
| mTuj1 | TAGACCCCAGCGGCAACTAT | GTTCCAGGTTCCAAGTCCACC |
| mT | CTCGGATTCACATCGTGAGAG | AAGGCTTTAGCAAATGGGTTGTA |
| mFlk1 | GGGTCGATTTCAAACCTCAATGT | AGAGTAAAGCCTATCTCGCTGT |
| mGata4 | CCCTACCCAGCCTACATGG | ACATATCGAGATTGGGGTGTCT |
| mGata6 | TTGCTCCGGTAACAGCAGTG | GTGGTCGCTTGTGTAGAAGGA |
| mCk18 | CAGCCAGCGTCTATGCAGG | CTTTCTCGGTCTGGATTCCAC |
| mCk19 | GGGGGTTCAGTACGCATTGG | GAGGACGAGGTCACGAAGC |
| mId1 | AGGTGAACGTCCTGCTCTAC | GTCCCGACTTCAGACTCCG |
| mId2 | ATGAAAGCCTTCAGTCCGGTG | AGCAGACTCATCGGGTCGT |
| mMsx1 | GCGCCTCACTCTACAGTGC | CTCTGGACCCACCTAAGTCAG |
| mMsx2 | AAGGCGAAAAGACTGCAAGAG | GCAGGGGTGAGTTGATAGGG |
| mWnt3 | TGGCTACCCAATTTGGTGGTC | CTTCACACCTTCTGCTACGCT |
| mDkk1 | CTCATCAATTCCAACGCGATCA | GCCCTCATAGAGAACTCCCG |
| mAxin2 | TGACTCTCCTTCCAGATCCCA | TGCCCACACTAGGCTGACA |
| mMyc | TCTCCATCCTATGTTGCGGTC | TCCAAGTAACTCGGTCATCATCT |
| mGAPDH | TGACCACAGTCCATGCCATC | GACGGACACATTGGGGGTAG |
| 1. **Primers for ChIP-qPCR** | | |
| Name | Forward primer （5′ to 3′） | Reverse primer （5′ to 3′） |
| ChIP-Id1-BRE1 | TTCAGACGCTGACACAGACC | CCTCTTGCAGAAAGGTTCCA |
| ChIP-Id1-BRE2 | TCTAACGGTCTGAGCCGCTTGTTC | CTACCCTGGATTTGCAGCCTCTTG |
| ChIP-Id1-3U | TTGGGAAAGCGGGTCACTGGA | TTGGGAAAGCGGGTCACTGGA |
| ChIP-Sox2N2 | CCAGTTAACAAGGGCATTCTCC | GCGAGAACTAGCCAAGCATC |
| ChIP-Sox2N1 | CCAAACTGGGGAGCAACTTA | TACATTGGAGTCCGGATGGT |
| ChIP-Pax6 | CTAGATGAGCAGTGAGGGC | CAGCTGCTCTGATTAAGATG |
| Control-Pax6 | GTGAGCCAGCCACATGATG | GTACATCTTTAGGCCTTCGG |
| ChIP-Sox2 | TTTGGAACCCACAGTTGACA | CATTCCGAGGAAGAGCAGAC |
| Control-Sox2 | TAACTCCACCATCCGGACTC | AAAGACCCCGAGGACTTACG |
| ChIP-Zfp521 | GGCATCGATGGAGAAAAAG | CATGCAATGGTATGCTAAAG |
| Control -Zfp521 | CGGGATCCCTGCCAACTGT | CTCTTTCTCCCTCCACCACC |
| ChIP-Gata4 | GTGGAGTGCTCAGCTCTTGG | GTTACCGGCCGGCCTATCT |
| Control-Gata4 | GATCTCCAGGTTACACCAC | CTTGTTGCTAGAGATGATGC |
| ChIP-Myc | CTAGGGATTGGTGGCTCTTG | GTAGCGTGTATAAGGTGCTC |
| Control-Myc | GCCTGTTGCTCAGATGCAG | GCTGGTGGATAGATCTCTTG |
| ChIP-Dkk1 | GCTAGGCTCACAATCAAGT | CTGATAGCTTCAAGATGTCC |
| Control-Dkk1 | CATCCTGATGATGGCAGTA | ATGATGTAAGACTGTGCCGA |
